# Growth dynamics, skeletochronology, and histovariability of the theropod dinosaur *Berthasaura leopoldinae*

**DOI:** 10.1002/ar.70110

**Published:** 2025-12-15

**Authors:** Geovane Alves de Souza, Marina Bento Soares, Luiz Carlos Weinschütz, Alexander Wilhelm Armin Kellner

**Affiliations:** ^1^ Programa de Pós‐graduação em Zoologia, Museu Nacional Universidade Federal do Rio de Janeiro Rio de Janeiro Brazil; ^2^ Laboratório de Sistemática e Tafonomia de Vertebrados Fósseis, Departamento de Geologia e Paleontologia, Museu Nacional Universidade Federal do Rio de Janeiro Rio de Janeiro Brazil; ^3^ Centro Paleontológico da Universidade do Contestado (CENPALEO) Universidade do Contestado Mafra Brazil

**Keywords:** age inferences, bone histology, Ceratosauria, growth rates, three‐front model

## Abstract

Here we present the bone histology of *Berthasaura leopoldinae* MN 7821‐V, a basal abelisauroid from the Late Cretaceous of Southern Brazil, and discuss the impacts of multi‐element sampling in ceratosaurs, including its biological implications. Femur, tibia, and fibula from the holotype were sectioned for this study. Age was estimated using LAG retro‐calculation and cyclical growth marks count. The cortex of the femur and tibia consists predominantly of parallel‐fibered tissue mixed with regions of fibrolamellar bone, which suggests slow osteogenesis similar to that inferred for abelisauroids rather than ceratosaurids and most theropod dinosaurs. In contrast, the fibula is predominantly composed of a fibrolamellar complex. Whereas the femur is primarily filled by longitudinally oriented vascular canals, with a band of plexiform, the tibia is primarily of longitudinal with a reticular pattern, and the fibula is exclusively of a simple longitudinal network with primary and secondary osteons. The bone cortices are commonly interrupted by lines of arrested growth (LAG) in the femur and tibia, and LAGs plus *annuli* in the fibula, indicating that the bone growth was periodic. Outer circumferential layers of lamellar bone were not recorded in any of the sampled elements. In addition, the thickness of the growth zones was consistent in the femur and tibia; that, in combination with absent outer circumferential layers, suggests that MN 7821 was a subadult that did not reach sexual maturity before death. This corroborates a previous ontogenetic assessment inferred based on morphology. Inter‐elemental variations in bone matrix type and growth record were observed in *Berthasaura*, showing that such discrepancy would impact metabolic inferences based on single bones. In addition, the particularly narrowing growth zones from the femur of MN‐7821‐V provided higher values of retro‐calculated LAG in comparison to the tibia and fibula. Based on this histovariability observed in the different elements of *Berthasaura*'s skeleton, our work also pointed to an underlying bias concerning LAG retro‐calculation based on metric extrapolations from a single bone, which reinforces the importance of working with a multi‐elemental sample.

## Graphical abstract

Osteohistological sampling on different bones of theropod dinosaur documents discrepant age record, growth, and metabolism. This could result unprecise paleobiological inferences if samplings are based on single bones. However, multi‐bone sampling can attenuate these discrepancies, helping to infer growth dynamics and physiology of these extinct dinosaurs.

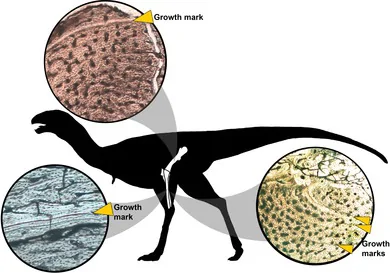

## 1 INTRODUCTION

Bone histology of extinct organisms can provide inferences on growth dynamics and ontogeny (e.g., Horner et al., 2000), thermophysiology (e.g., Ricqlès, 1974), taxonomy (e.g., Horner & Goodwin, 2009), and ecology (e.g., Tutken et al., 2004). Bone histology can validate or refute morphological ontogenetic assignment of fossil organisms (e.g., Farias et al., 2024) and thus may help to determine whether variation observed within a fossil assemblage correlates with development. Besides, because histology focuses on tissues, it can offer relatively abundant data about development pathways, age, and timing of maturation based on a few isolated bones, being suitable for the taxa with a poor fossil record, such as theropod noasaurids and their close relatives.

Bone microstructure varies within the cross‐section, within the diaphysis, across the skeleton, or from individual to individual. This variation evokes the concept of histovariability, that is, the microstructural variation in a given bone or histological section influenced by ontogeny, phylogeny, biomechanics, and environmental factors (Padian & Lamm, 2013). Variation may involve differences in growth mark count, predominant bone matrix type, vascularization pattern, degree of secondary ossification (Cullen et al., 2014; de Margerie et al., 2002), among others. Inter‐element variation has been extensively documented in reptiles, including dinosaurs (e.g., Cullen et al., 2014; Horner & Padian, 2004). Histovariability plays an important role in histological studies, notably in analyses of poorly preserved skeletons or isolated bones. Histological sampling of a bone section captures only a fraction of an individual's microanatomy, thereby underestimating ontogenetic or metabolic inferences. Only multi‐element sampling can adequately cover inter‐skeletal histovariation and provide a comprehensive microanatomical framework for paleobiological inferences. Unfortunately, few fossil species have skeletal microstructure preserved sufficiently to allow such analyses.

The discovery of the Cretaceous site named *Cemitério dos Pterossauros* (Pterosaur Graveyard) Quarry in Paraná State (Southern Brazil) has revealed hundreds of fossil bones. Two tapejarid pterosaur species, one stem‐Acrodonta lizard, and two theropod dinosaurs, *Vespersaurus paranaensis* and *Berthasaura leopoldinae*, have been described from this location (de Souza et al., 2020; de Souza et al., 2021; Kellner et al., 2019; Langer et al., 2019; Manzig et al., 2014; Simões et al., 2015). Among these fossils is the edentulous noasaurid *B. leopoldinae* MN 7821‐V, which consists of a well‐preserved and almost complete skeleton of a small‐sized individual. The completeness of the holotype MN 7821‐V elucidated many aspects of the anatomy of noasaurids, reporting unprecedented traits for Ceratosauria.

Here we combined osteohistological descriptions and skeletochronology of the femur, tibia, and fibula of MN 7821‐V to (1) describe the microanatomy of its appendicular bones; (2) assess the histovariability within the hindlimb bones; (3) assess the ontogenetic stage of the individual; and (4) compare *Berthasaura*'s microstructure and growth dynamics with other ceratosaurs.

## 2 MATERIALS AND METHODS

### 2.1 Histological sampling

The left femur, left tibia, and left fibula of *Berthasaura* (MN‐7821‐V, Figure 1) were chosen because these appendicular elements were sampled in previous paleohistological studies on other Noasauridae (de Souza et al., 2020; Lee & O'Connor, 2013; Wang et al., 2017), which allow for comparisons.

**FIGURE 1:**
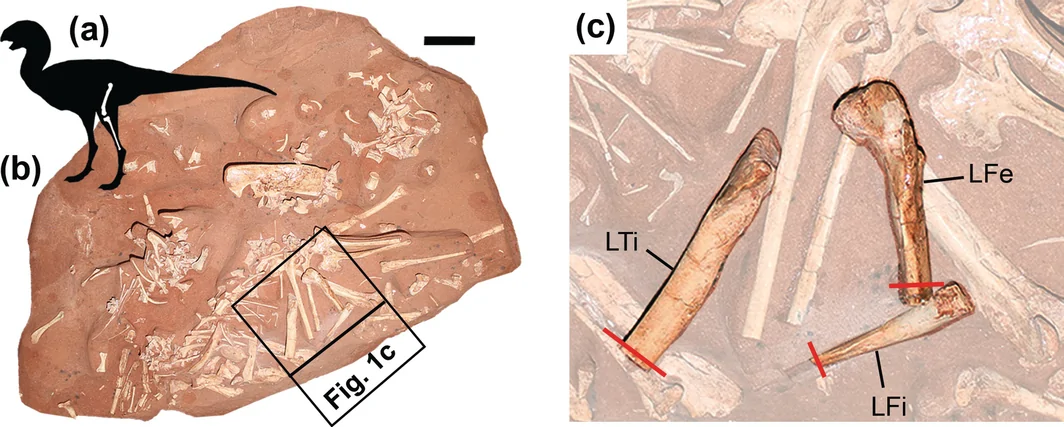
*Berthasaura leopoldinae*; multi‐element histological sampling. Silhouette of *Berthasaura leopoldinae* (a), photograph of the block MN 7821 V (holotype) (b), and bones sectioned in the study (c). LFe, left femur; LFi, left fibula; LTi, left tibia. Scale bar = 5 cm.

The methodological procedures were carried out in the Laboratório de Paleohistologia of the Museu Nacional, Universidade Federal do Rio de Janeiro (UFRJ), Rio de Janeiro, Brazil. The sections were made in midshaft because this is the most suitable for assessing the best growth record (Stein & Sander, 2009), and at standardized regions, approximately mid‐way along the proximal half of each bone's length (Figure 1c). Regions with muscle scars were avoided to not overestimate the circumferential and diameter measures.

The paleohistological protocol follows de Souza et al. (2020) and de Souza et al. (2023), which is in turn adapted from Lamm (2007). Fossils were covered by epoxy resin Rp031 near the section level to protect the specimens against the saw vibration. Sectioning was done by a precision router (Dremel® Micro 8050‐N/18, Racine, WI, USA) after the resin hardened. Bone fragments were then soaked in epoxy resin to produce resin blocks. One side of the resin blocks was polished using a lap wheel Politriz Aropol® VV200‐PU and sandpaper Norton Saint‐Gobain® T277 with P100, P600, and P1200 lines/inch sequentially. The opposite block surface was glued on a histological slide using epoxy resin, whereas the other side was thinned in the Buehler PetroThin® thin section machine and polished using the same sandpaper series.

### 2.2 Microstructural imaging and histological descriptions

Histological slides were numbered and deposited in the Museu Nacional collection. Histological slides were analyzed using a Stereomicroscope Zeiss 4 to obtain panoramic views of the cross‐sections (Figures 2a, 3a, 4a, and Supporting Information Data S1–S3) and an Axioscope 5 transmitted light microscope with conventional white‐light reflection and transmitted circularly polarized light with 5×, 10×, or 20× objectives. Images were acquired with an Axiocam 208 Color (8MP, 1/2.1″) video camera using ZEN® 3.4 imaging software and prepared for publication in Photoshop CS6 software.

**FIGURE 2:**
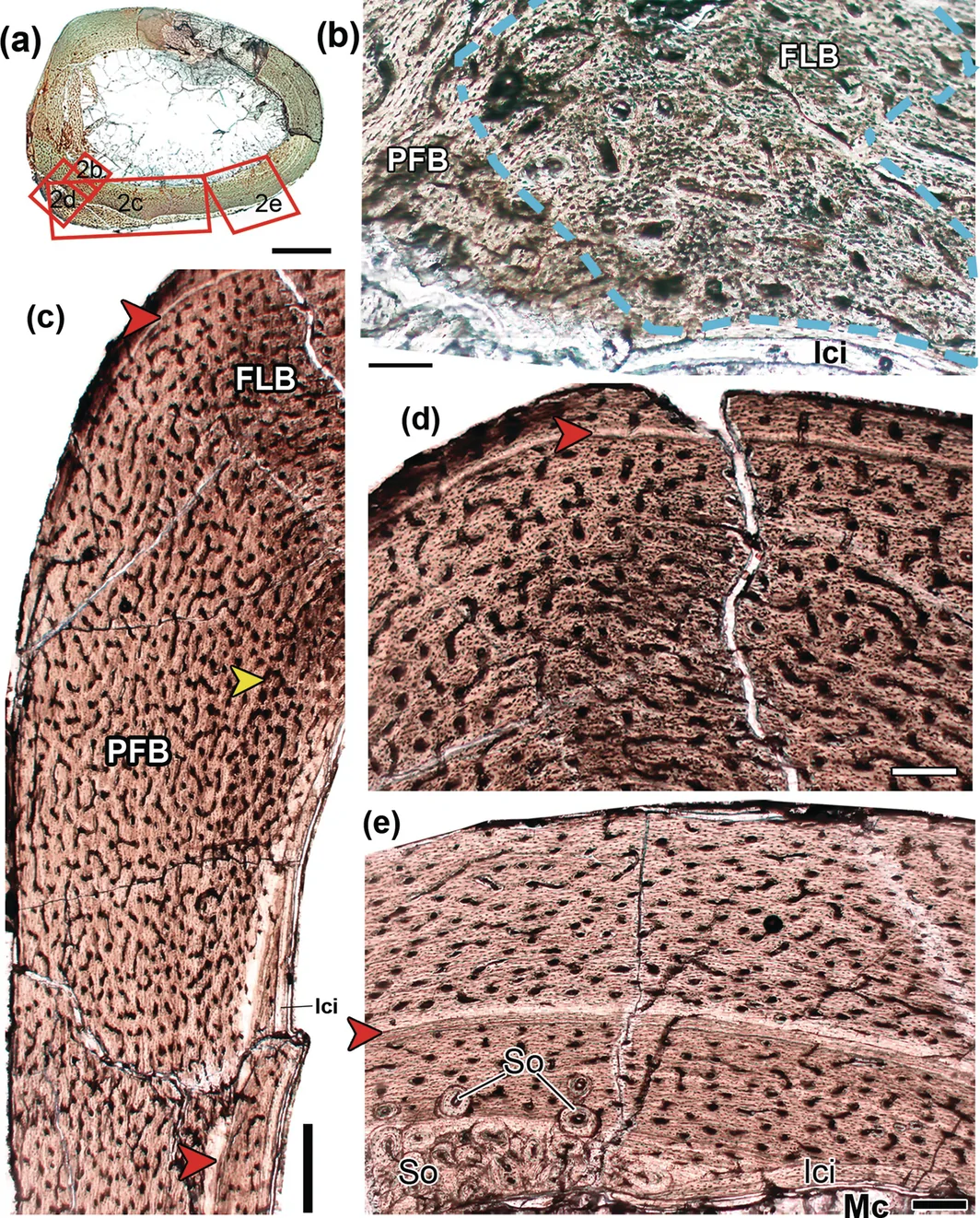
*Berthasaura leopoldinae*; femur histology. Femoral whole cross‐section (a). Two types of bone matrices (woven and parallel‐fibered) in detail (b). Femoral cortex showing distribution of woven and parallel‐fibered bone matrices (c). Subperiosteal cortex showing outermost LAG in detail (d). Perimedullary cortex in detail showing lamellae circumferentiales internae, innermost Line of Arrested Growth (LAG), and secondary ossification (d). lci, lamellae circumferentiales internae; Mc, medullary cavity; Oc, osteocyte clusters; PFB, parallel‐fibered bone; So, secondary osteons; FLB, fibrolamellar bone. Blue dashed line indicates fibrolamellar tissue extent. Red arrows denote LAGs. Yellow arrow denotes vascular cycle bounding polishing line (internally). Scale bars: a = 2 mm; c = 500 μm; b, d, e = 100 μm. 25X magnification in c and 100X in b, d and e.

**FIGURE 3:**
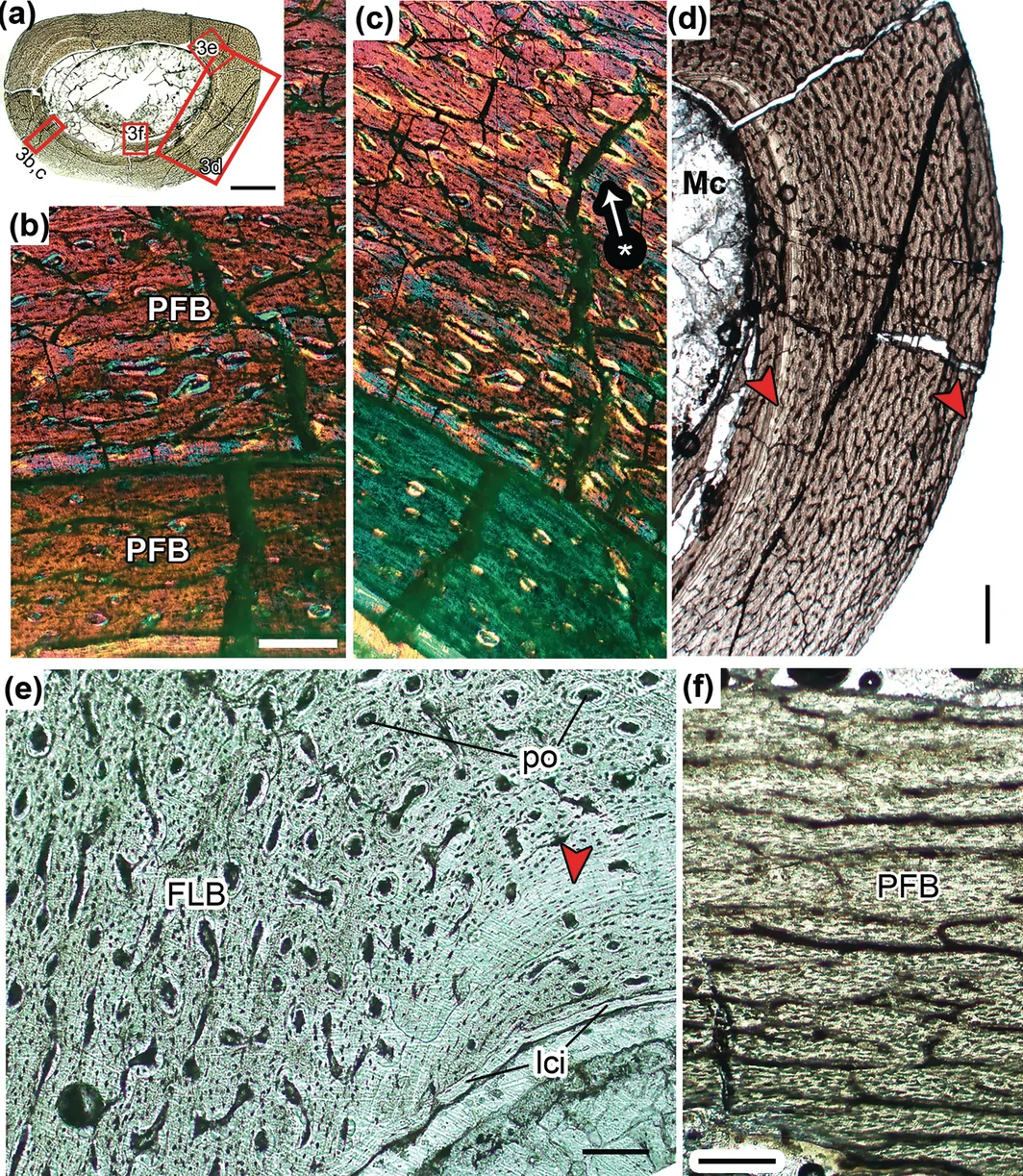
*Berthasaura leopoldinae*; left tibia histology. Whole cross section of tibia (a). Cortex under cross polarized light at 0° (b) and 45° (c) degree of rotation. Cortex showing the two Lines of Arrested Growth (LAGs) (d). Perimedullary cortex showing fibrolamellar tissue, lamellae circumferentiales internae, and innermost LAG (e). Perimedullary cortex comprising of plexiform vascular network devoid of primary osteons (f). Abbreviations: FLB, fibrolamellar bone; lci, lamellae circumferentiales internae; Mc, medullary cavity; PFB, parallel‐fibered bone; Po, primary osteon. Red arrows denote LAGs. Asterisk denotes areas of distinct collagen fiber orientation within the parallel‐fibered tissue. Scale bars: a = 2 mm; b and c = 200 μm; d = 500 μm; e and f = 100 μm. 25X magnification in d, 50X in b and c; and 100X in e and f.

**FIGURE 4:**
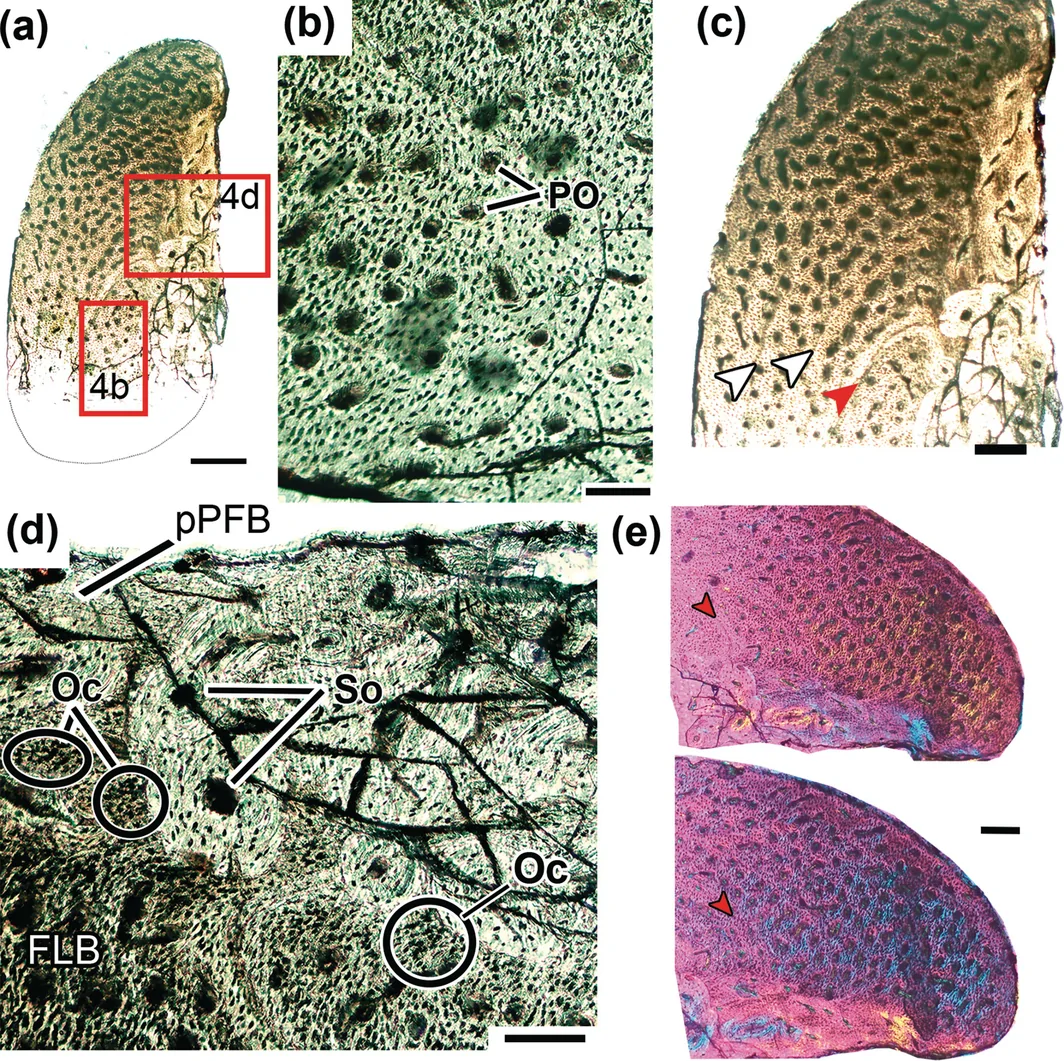
*Berthasaura leopoldinae*; Fibula histology. Whole cross section of fibula (a). Cortex in detail showing globular osteocytes surrounding primary osteons (b). Cross section of fibula showing the cyclical growth marks (c). Cortex in detail showing clusters of osteocyte lacunae and bone remodeling represented by large secondary osteons (d). Fibular section under circular polarized light (0° and 45° rotation) (e). Abbreviations: FLB, fibrolamellar bone; pPFB, poorly‐cellular parallel‐fibered bulk of bone; Po; Primary osteons; Oc, Osteocyte lacunae clusters; So; secondary osteons. Red arrow denotes Line of Arrested Growth (LAG). White arrows denote *annuli*. Scale: a = 0.4 mm; d, e = 200 μm; b, c = 100 μm. 50X magnification in a, c, and e; 100X in b and d.

The nomenclature follows Nomina Histologica Veterinaria (1994). The definition of the histological structures follows Padian and Lamm (2013), Buffrénil et al. (2021) and references cited herein. Cyclical growth marks are the expression of a biochronological rhythmicity that can be found in all skeletal elements (Castanet et al., 1993). Some cyclical growth marks (e.g., vascular cycles and polishing lines) have variable periodicity (e.g., Woodward, 2019). Others, such as lines of arrested growth (LAGs) and *annuli*, are cyclical growth marks formed most often in response to endogenous seasonal rhythms (Castanet et al., 1993; Castanet & Smirina, 1990; Francillon‐Vieillot et al., 1990), being annual indicators (Chinsamy, 1997; Horner et al., 2000). The LAGs consist of lineae cementales (=cement lines) that could run nearly the entire cross‐section and indicate a complete cessation in bone growth, whereas *annuli* are layers of lamellar or parallel‐fibered bone lacking linea cementalis, which denote a temporary decrease in growth rate (Peabody, 1961). It is assumed that the transition from growth acceleration to deceleration, reflected by attenuated LAG/*annuli* spacing, may coincide with the onset of reproductive maturity (Lee & Werning, 2008). Lamellae circumferentiales internae (=endosteal lamellae, inner circumferential layers; Buffrénil et al., 2021) are layers of parallel‐fibered or lamellar tissues, variable in thickness, on the wall of the medullary cavity. The presence of lamellae circumferentiales internae reflects a cortical drift of the bone centroid and the medullary cavity during growth (i.e., perimedullary remodeling), and is commonly regarded as an indicator of bone maturity (Goldman et al., 2009; Padian & Lamm, 2013). When an individual reaches somatic maturity, growth ceases, and a poorly vascularized, commonly acellular, lamellar bone with a compressed pack of LAGs, the lamellae circumferentiales externae (=outer circumferential lamellae, external fundamental system), are deposited in the outermost cortex (Francillon‐Vieillot et al., 1990; Woodward et al., 2011).

Microanatomical descriptions follow the three‐front model approach (Mitchell & Sander, 2014), which is a developmental framework designed to explain the long bone diaphyseal histology (originally for sauropods, but later adopted for other amniotes). The model conceptualizes three radially progressing fronts responsible for bone formation: apposition front, Haversian substitution front, and resorption front. The apposition front encompasses the deposition of new bone onto the periosteal surface, and its rate of formation is determined by the type of primary bone tissue. The resorption front marks the expansion of the medullary cavity via endosteal resorption, progressing to maintain optimal cortical thickness. Finally, the Haversian substitution front describes the replacement of primary bone in the cortex by secondary osteons, moving from the innermost cortex outwards.

### 2.3 Skeletochronology

Theropod dinosaurs exhibit hollow bones with wide medullary cavities. Both cortex remodeling and the medullary cavity expansion obscure the earliest growth marks and lead to underestimated ages. Thus, we used the theoretical “extrapolation” models proposed by Horner and Padian (2004) and modified by Lee and Werning (2008) to estimate the inter‐LAG distances and subsequently retro‐calculate the earliest missing growth record. These models use different distances between adjacent cyclical growth marks and extrapolate to the radius of the medullary cavity (missing growth record). Details of the methods are provided in Supporting Information.

First, the lost bone tissue (i.e., medullary cavity plus innermost preserved growth zone) is measured. The maximum diameters in the X and Y axes of the medullary cavity are calculated, and the intersection point is assumed to be the bone centroid. The distance between the centroid and innermost preserved LAG is measured to obtain the missing growth record (Tables S1 and S2).

Second, size or weight at birth is the smallest mass/size in the growth curve, adds important biological information and realism by constraining the estimation at the lower end (Grady et al., 2014). Then, we discounted the neonatal femoral/tibial radius to the missing cortices. We assumed the femur/tibia radius of the hatchling as ca. 2 mm based on egg estimation and size of a larger, presumably mature individual of *Berthasaura* (personal communication based on unreported femoral fragment CP.V 8662). Thus, the missing growth record assumed here is the missing + partially eroded innermost cortex radius (average) subtracted by the hatchling radius.

Third, to infer how many LAGs could be formed after the medullary cavity expansion, the missing annual intervals are calculated using different theoretical models: “Mean”, “Maximum”, “Penultimate”, “Incremental Factor” and “Parabolic” (Horner & Padian, 2004). In the “Mean” model, the average thickness of the preserved growth intervals is calculated and used as a parameter to reconstruct the missing LAGs toward the medullary cavity. The “maximum” model assumes the width of the thicker growth interval—commonly the innermost preserved interval, as a parameter to reconstruct the erased growth record. The “penultimate” model assumes the thickness of the penultimate deepest LAG interval as the parameter (if the innermost interval is peculiarly narrower). The Incremental Factor Model considers a mean percentage increase in the width of LAG intervals, moving centripetally, whereas in the Parabolic Model, hypothesizing that LAG intervals were maximal at the innermost preserved LAG, the intervals of the series progressing centrifugally were applied to the missing centripetal series, mirrored.

The inter‐LAG spaces vary depending on the positions within the sampled bone cross‐section, which can result in substantially different age estimations. We counted the LAGs and measured the distances of successive parts of the cross‐section in each specimen (Table S1). We only counted the LAGs in the fibula (see discussion in Section 4.2. Histovariability within MN‐7821‐V forelimb bones). Depending on the level of the shaft, fibulae are composed of compacta and may lack a medullary cavity. Complete or partially obliterated fibular medullary cavities could result from the development of gender‐related medullary bone (Hübner, 2012; Prondvai, 2017), paleopathology (Griffin, 2018), or morphological simplification of the fibula to spindle‐like bone presenting a loose connection with the ankle (Botelho et al., 2016). Therefore, except in the case of paleopathology, the fibula is expected to preserve the most complete growth record. This could result from limited intracortical remodeling, especially in the perimedullary resorption, as it was inferred in anurans and lizards (Esteban et al., 1998; Leclair, 1990).

Bone microstructure, age estimations, and anatomy were used as proxies to determine maturity for the holotype of *Berthasaura* MN‐7821‐V.

## 3 RESULTS

### 3.1 Femur MN‐7821‐V histology

The femur is rounded‐triangular in cross‐section, with the ‘base’ of the triangle corresponding to the medial surface of the bone (Figure 2a; Figure S1). The lateral and posteromedial vertices of the cross‐section correspond to the longitudinal keels of the femoral shaft that separate the origin areas of the *M. femorotibialis internus* and *externus*, but no apparent Sharpey's fibers were observed in the cortex. The longitudinal neurovascular pattern is predominant in the femur (Figure 2b–e), grades to reticular, then longitudinal again toward the outer cortex, in the more densely vascularized areas (Figure 2c).

Two types of bone matrix can be recognized in the femur. In the anteromedial and posteromedial corners of the femur, the osteocyte lacunae are almost all globular and randomly oriented, in contrast to being flattened and parallel‐oriented (Figures 2b–d and 3e). Occasionally, they agglomerated to form dense osteocyte clusters lacking orientation (Figure 2b,d), which suggests that the collagen fibers were deposited without any predominant orientation in life. Such osteocyte clusters correspond to about 5% to 10% of the cross‐sectional area in the femur. This pattern is typical of the woven‐fibered bone. Primary osteons are rare, but they occur in both femora (Figure 2b). The combination of woven‐fibered matrix and primary osteons characterizes the fibrolamellar bone complex, a fast‐growing bone tissue commonly found in juvenile tetrapods and widespread in all ontogenetic stages in many dinosaurs (Bybee et al., 2006; Enlow, 1969; Enlow & Brown, 1957; Mitchell et al., 2017; Mitchell & Van Heteren, 2016; Ricqlès, 1969). Except for the anteromedial and posteromedial corners, the femoral cortex of MN 7821‐V is mostly composed of parallel‐fibered bone, a typical slower‐growing bone tissue, documented in the representatives of the abelisaurids and noasaurids (de Souza et al., 2020; Lee & O'Connor, 2013).

The cortex is interrupted by two LAGs in femur (Figure 2c–e). LAGs are bounded externally by a halo of poorly vascularized parallel‐fibered bone. The innermost LAGs and growth zones were partially eroded by the medullary cavity expansions, forming an innermost growth zone particularly narrow (Figure 2c–e; Tables S1 and S2). The growth zones between the first and second LAGs are thicker than the growth zones between the second LAG and the subperiosteal cortex (Figure 2c–e; Table 2; Table S1). The intermediate growth zone has a well‐defined polishing line (Figure 2c; Figure S1), which is an elevated band reflecting greater hardness than surrounding bone (Erickson, 2014; Sander, 2000). The polishing line in MN 7821‐V presents as a darker band of bone between the two LAGs. This cyclical growth mark can be seen in the slice by the naked eye. The polishing line is darker in the area of the cross section formed by fibrolamellar bone, brightening toward the region of parallel‐fibered bone. The polishing line is bounded externally by a vascular cycle (Figure 2e). Both polishing lines and vascular cycles comprise cyclical growth marks, although they do not reflect annual cycles (Curry, 1999; Klein & Sander, 2008; but see Sander, 2000 for an alternative interpretation). The outermost LAG (Figure 2c) was close to the outer bone surface (Table 2).

The medullary cavity is wide and occupies 45.9% of the cross‐sectional area of the femur (Table 1). Lamellae circumferentiales internae cover the medullary cavity. Neurovascular canals rarely cross through the lamellae circumferentiales internae.

**TABLE 1:** Measurements and histological features of the femur, tibia, and fibula of *Berthasaura leopoldinae* (MN‐7821‐V).

Bone	Circumference (mm)	Growth marks type and number	Lamellae circumferentiales interna	Medullary cavity (%)	Main type of primary bone
Femur	31.54	2 LAGs	Yes	45.9	PFB + FBL
Tibia	31	2 LAGs	Yes	38.9	PFB + FBL
Fibula	7	1 LAG + 2 annuli?	—	—	FLB + PFB*

*Note*: Circumferences were taken at the level of midshaft. In addition, lamellar bone was found within primary osteons, lamellae circumferentiales interna, and/or associated with cyclical growth marks in all bones of MN‐7821‐V.

Abbreviations: FLB, fibrolamellar bone; LAG, lines of arrested growth; PFB, parallel‐fibered bone; PFB*, poorly vascularized parallel‐fibered bone.

The secondary ossification is moderate and restricted to the lateral perimedullary cortex of the femur and did not exceed externally the innermost LAG. The Harversian substitution partially obscured the lamellae circumferentiales internae (Figure 2e). Secondary osteons vary in two orders of magnitude. There are no more than two osteonal generations.

Expansion of the medullary cavity eroded the growth record in the femur (Figure 2c,e). The pattern of LAG erosion and secondary ossification suggests that medullary cavities medially displaced (cortical drift) through the ontogeny of MN 7821‐V femur. The homogeneity of neurovascular canals through the cortex indicates that the bones did not change their blood supply near the time of death. The femur lacks lamellae circumferentiales externae, indicating that MN‐7821‐V was not fully grown at the time of death.

### 3.2 Tibia MN‐7821‐V histology

The tibia has a “D‐shape” in cross section, with the flattened margin corresponding to the contact surface with the fibula (Figure 3a; Figure S2). Sharpey's fibers were apparently absent. As in the femur, the tibia has two types of bone matrices, a predominant parallel‐fibered and a region of woven‐fibered tissue. Few primary osteons occur within the woven matrix, picturing a fibrolamellar bone. The fibrolamellar area occupies about 25% of the tibia (against 5% in the femur).

In the inner cortex of the tibia, the predominant pattern is characterized by a bone matrix that is highly birefringent in mass under cross‐polarized light (Figure 3b,c). This tissue is filled with ellipsoid to rounded osteocyte lacunae, interspersed with small regions of spindle‐shaped osteocyte lacunae (Figure 3b,c). Lacunae are not exactly parallel to each other; instead, they are oriented in waves. The combination of mass birefringence with flattened to globular, relatively parallel orientation of osteocyte lacunae indicates moderate collagen fiber organization. These traits depict the parallel‐fibered bone matrix.

Tibia has a complex optical behavior. First, the typical woven pattern is restricted to a bulk of woven bone at the medial aspect of the section, as in the femur. However, the perimedullary cortex in the remaining areas exhibits two optical behaviors. The inner growth zone is mostly occupied by ellipsoid to globular osteocyte lacunae with a small portion of spindle‐shaped lacunae. Despite osteocyte lacunae not being as flattened as in typical parallel‐fibered bones, the inner growth zone is birefringent under cross polarized light, which could indicate less organized or obliquely oriented collagen fibers of a parallel‐fibered matrix. In the outer cortex, the osteocyte lacunae have alternating bands of globular and flattened osteocyte lacunae, which suggests alternation between moderately disorganized/oblique fibers composing the parallel‐fibered matrix and orthogonal fibers of a typical parallel‐fibered matrix. These bands show an overall monorefringence under cross polarized light (Figure 3b,c), which could suggest more disorganized collagen fibers typical of woven matrices. It is important to note that if collagen fibers are transversely sectioned at their main longitudinal axes, even in parallel‐fibered matrices, they display a dark aspect (thus isotropic) under cross polarized light (Prondvai et al., 2014). Thus, parallel‐fibered bone matrices can show monorefringence in cross polarized light, which is inferred to occur in the outer growth zone of tibia MN 7821 V. In addition, the outer zone is occasionally intercepted by lenses of bright and birefringent bone matrix with spindle‐shaped osteocyte lacunae. These lenses typify portions of typical parallel‐fibered bone matrices. Differences in the shape of osteocyte lacunae and optical behavior between the growth zones of tibia could result from alternation between static and dynamic osteogenesis (Prondvai et al., 2014), different degrees of fiber orientation, or both.

The cortex is interrupted by two LAGs in the tibia (Figure 3b–e). Only the innermost LAG is bounded externally by a halo of poorly vascularized parallel‐fibered bone. The outer LAG consists of a linea cementalis devoid of parallel‐fibered bone. The innermost LAGs and growth zones were partially eroded by the medullary cavity expansions, forming an innermost growth zone particularly narrow (Figure 3d; Tables S1 and S2), similar to the femur. The growth zones between the first and second LAGs are thicker than the growth zones between the second LAG and the subperiosteal cortex (Figure 3d; Table 2). In the tibia, the intermediate growth zone preserves a vascular cycle bounding a well‐defined polishing line, similar to the presumably equivalent growth zone in the femur. The outermost LAG (Figure 3d) was close to the outer bone surface (i.e., subperiosteal cortex).

**TABLE 2:** Width of the growth zones, growth record, and age estimation of the femur, tibia, and fibula of *Berthasaura leopoldinae* (MN‐7821‐V).

Bone	Growth zones width average from the innermost to the outermost cortex (mm)	CGM number	Retro‐calculated LAGs	Age estimation (years)
Femur	0.50*; 0.79; 0.13*	2	2	4
Tibia	0.49*; 1.56; 0.07*	2	1	3
Fibula	0.58? 0.11; 0.31; 0.52	3	—	3

*Note*: Growth zones of the fibula were not used to retro‐calculate the missing growth record. Asterisks (*) denote growth zones that deposition was interrupted by premature death of the specimen or medullar cavity expansion. Growth zones listed from innermost to outermost ones. The innermost values account for the distance between bone centroid and innermost cyclical growth mark.

Abbreviations: CGM, cyclical growth marks (specifically annule and LAGs); LAGs, line of arrested growth.

The tibia exhibited lamellae circumferentiales internae but lacks secondary ossification. The medullary cavity occupies 38.9% in the tibia (Table 1). The tibial cortex comprises mostly reticular canals (Figure 3b–e), with some areas of plexiform neurovascular network (Figure 3f). Expansion of the medullary cavity eroded the growth record in the tibia. The tibia lacks lamellae circumferentiales externae, as in the femur, corroborating that MN‐7821‐V was not fully grown at the time of death.

### 3.3 Fibula MN‐7821‐V histology

The fibula is “D‐shaped” in cross section (Figure 4a; Figure S3). The flattened edge of the “D” corresponds to the surface of the fibula that contacts the tibia in life. The anterior edge of the section was lost during histological slice preparation. As in the femur and tibia, no Sharpey's fibers were observed in the cortex.

The sampled level of the fibular cortex lacks a medullary cavity and, as a consequence, it has no lamellae circumferentiales internae. Osteocyte lacunae are predominantly globular (Figure 4b–d). This pattern differs from the femur and tibia, in which ellipsoid, flattened, and spindle‐shaped osteocyte lacunae are abundant. Most osteocyte lacunae are positioned concentrically around the neurovascular canals and primary osteons. The osteocyte lacunae that are not associated with neurovascular canals lack predominant orientation. Clusters of osteocyte lacunae occur over almost all cortex (Figure 4d). The combination of haphazardly oriented and globular‐shaped osteocyte lacunae, sometimes disposed of in clusters, pictures the woven‐fibered bone matrix. Under polarized light, areas of bright and anisotropic tissue occur only in the lamellae that surround primary osteons (formerly of lamellar or parallel‐fibered bone), whereas most of the cortex is monorefringent (Figure 4e). Thus, the woven‐fibered matrix is predominant over the parallel‐fibered matrix in the fibula of MN 7821 V. This contrasts with the femur and tibia here studied, which have predominantly parallel‐fibered bone.

The neurovascular network of the fibula comprises simple longitudinal canals grading to a weak reticular pattern posteriorly. In the subperiosteal cortex, the neurovascular canals open to the outer bone surface, suggesting a high apport of blood supply and intense bone growth through accretion until the time of death. Primary osteons are abundant over the most cortex, except in the margin that contacts the tibia (Figure 4b–d). The combination of woven‐fibered matrix and primary osteons pictures the fibrolamellar complex (Ricqlès, 1974).

Three cyclical growth marks can be recognized in the fibula (Table 1; Figure 4c). These marks do not persist over the entire fibular cross section. Instead, they seem to obliterate toward the anterior side. These cyclical growth marks do not perform clearly circular trajectories, as seen in other long bones. This is typical of asymmetrical growth. The innermost cyclical growth mark has a linea cementalis, indicating full interruption of bone deposition, and therefore is interpreted as a LAG (Figure 4c). Examinations through cross‐polarized light reveal that the two outermost growth marks are almost invisible, presenting only as poorly vascularized bone belts, lacking linea cementalis (Figure 4e). This indicates that the cortex reduced bone deposition across these growth marks, rather than underwent a fully arrest of osteogenesis. These features typify *annuli* (Figure 4e; Supporting Information).

The medial margin of the fibula has a bulk of primary tissue consisting of poorly vascularized lamellar bone. This region is intercepted by secondary osteons (Figure 4c). No more than two osteonal generations occur in the medial margin of the fibula. These traits indicate that this region was subjected to some degree of bone remodeling near the contact with the tibia.

### 3.4 Apposition, resorption and Harversian substitution fronts of MN 7821‐V

The *Berthasaura* specimen MN 7821‐V is an immature, probably an early subadult individual. The thick layers of highly vascularized, mostly parallel‐fibered bone with a few areas of fibrolamellar tissue indicate that the apposition front was advancing rapidly in MN 7821‐V. In addition, the apposition front was similar to *Masiakasaurus* and *Vespersaurus*, but slower than *Ceratosaurus* and most theropods because of the LAGs interrupting parallel‐fibered bone apposition (de Souza et al., 2020; Lee & O'Connor, 2013; Sombathy et al., 2025). Appositional activity appears to be outpacing resorption and remodeling of the bone cortex, as evidenced by the outward homogeneity in vascular canal diameters and density, the relatively thin lamellae circumferentiales internae, and the poorly developed (femur and fibula) or absent (tibia) secondary ossification (Figures 2d,e, 3d,e, and 4a–e). This enables the increase in cortical thickness and the growth of the individual itself. The presence of thin lamellae circumferentiales internae (and the presence of resorption cavities in the fibula) indicates that the resorption front was still active, but slowing down, even before developing intensive bone remodeling. The absent Haversian substitution front in the tibia and a few scattered secondary osteons in the perimedullary cortex of the femur and medial margin of the fibula indicate that the resorption front progressed more rapidly than the Haversian front, preventing the formation of extensive Haversian tissues.

### 3.5 Skeletochronology

We retro‐calculated the missing LAGs using all five models proposed by Horner and Padian (2004), but only “Maximum”, “Penultimate” and “Parabolic” models fitted reasonably well with our data. As the cortices prior to the innermost LAGs were significantly eroded, the “Maximum” model (which considers the width of thicker growth zone – commonly the innermost inter‐LAG space – as a proxy to reconstruct the missing LAGs) is equal to the “Penultimate” model. Also, Horner and Padian (2004) mention that the Parabolic Model likely results in an underestimation of the growth rate in the early years of life. Because MN 7821‐V preserved peculiarly narrow outermost and innermost growth zones, the average growth zone thickness is underestimated, and we opted to discard the “Mean” model. Given the low number of inter‐LAG spaces and anomalously discrepant growth zone thicknesses due to incomplete innermost and outermost growth zones of MN‐7821‐V, the “Incremental factor” model was also discarded due to the unrealistic results. Thus, we considered the “Maximum” and “Penultimate” models because they best fit our data.

One (tibia) and two (femur) cyclical growth marks were retro‐calculated based on “Maximum” and “Penultimate” models to MN‐7821‐V (Table 2; Table S3). The number of preserved growth marks plus retro‐calculated growth marks totaled four (femur) and three (tibia) years old at the time of death for MN‐7821‐V (Table 2). Therefore, the minimum age for MN‐7821‐V is 3 years old, and the maximum is four.

## 4 DISCUSSION

### 4.1 General bone microstructure and growth pattern

The microstructure of MN‐7821‐V allows us the comparison with taxa previously studied and provides insights about the life history of the subadult individual of *Berthasaura*. The presence of LAGs in the cortex of the femur, tibia, and fibula of MN‐7821‐V indicates a cyclical growth strategy, as in non‐neornithine theropods (D'Emic et al., 2012; Erickson et al., 2009; Horner & Padian, 2004; Rolando et al., 2021; Varricchio, 1993), including ceratosaurs (Baiano & Cerda, 2017; Canale et al., 2016; Evans et al., 2015; Lee & O'Connor, 2013). *Berthasaura*'s microstructure, however, differs from most other theropods by having femur and tibia with dense packs of parallel‐fibered bone type, predominantly in the perimedullary cortex, which is indicative of lower rates of osteogenesis, and the outer cortex formed by woven‐fibered bone matrix (mainly in medial corners of femur and tibia) with some degree of osteocyte orientation. The parallel‐fibered pattern is widespread among noasaurids (de Souza et al., 2020; Lee & O'Connor, 2013; Martinelli et al., 2019), and abelisaurids (Canale et al., 2016; de Souza et al., 2020), and is regarded to have evolved independently in basal members of the bird‐line theropod lineage (Erickson et al., 2009).

### 4.2 Histovariability within MN‐7821‐V forelimb bones

Only a few distinctive histological variations were recognized between the femur and tibia of *Berthasaura*. The fibula, in contrast, exhibited the most discrepant microstructure among the sampled bones, revealing an already expected histovariability within the hindlimb bones. The theropod fibula is subject to lesser intense biomechanical stresses of the body weight compared to the femur and tibia, which impacts microstructural traits, such as the degree of bone remodeling, vascularization, and growth trajectory. Although histovariability is expected to be common, it was observed in just a few archosaur taxa (e.g., Horner et al., 1999; Sander, 2000; Padian et al., 2001; Sayão, 2003; Eleutério et al., 2015, and references herein). Therefore, the most important sources of histovariability in *Berthasaura* are the type/count of growth marks and the type of bone matrix.

*Annuli* and LAGs vary depending on the element. Femur and tibia have two fully formed LAGs, and fibula has one LAG and two *annuli*. This suggests that femur and tibia were subjects of fully growth interruption during development, while fibula varied from episodes of total bone growth interruption (in early ontogeny; LAG) to decelerating osteogenesis (in the second and third years of life; *annuli*). This variation of growth within fibula may indicate that MN‐7821‐V experienced an explosive phase of growth (i.e., narrow spacing between cortical growth marks deep in the cortex, followed by externally more widely spaced marks, followed again by more narrowly spaced marks; e.g., D'Emic et al., 2023). Among theropods, this phase is commonly observed in elements that have suffered less dense cortical remodeling and medullary expansion, such as ribs and fibulae (e.g., D'Emic et al., 2023). Alternatively, theropod fibulae increase in width asymmetrically during development. Such asymmetry correlates with differential bone deposition on one side of the cortex once an initial bone model has been formed. *Annuli* of the MN 7821‐V fibula lie only on one side of the cortex, which could be a result of asymmetrical growth rather than explosive growth.

Regarding growth marks count, the fibula of MN‐7821‐V preserves three cyclical growth marks (CGM = LAG or *annuli*), whereas the femur and tibia have two CGM. This variation of the growth record is similar to what has been observed in other theropods (Horner et al., 1999; Horner et al., 2000), sauropods (Waskow & Sander, 2014), and ornithischians (e.g., Horner et al., 2000). In smaller‐bodied theropods (less than 1 ton) and larger‐bodied individuals in which the remodeling did not impact the primary growth record, the number of cyclical growth marks varies only slightly between elements of the same individual, contrasting with the higher level of inter‐elemental variation in LAG count seen in larger‐bodied theropods such as tyrannosaurids and carcharodontosaurids (Cullen et al., 2014; Cullen et al., 2020). In this sense, the inter‐elemental variation of MN‐7821‐V is similar to that seen in other small‐bodied theropods. Intra‐ and inter‐skeletal variations in all these theropods are regarded as a result of different rates of periosteal deposition and local conditions of growth (pathology, starvation, muscle attachments, weighting stress; Horner et al., 1999; Horner et al., 2000; Cullen et al., 2014; Cullen et al., 2020). The same can be assumed about sampled bones of *Berthasaura*, which have distinct biomechanical functions (despite the apparent absence of Sharpey's fibers in *Berthasaura*'s sections) and/or developmental pathways.

Fibulae of small‐bodied theropods often preserve equal or more growth marks in comparison to other sampled bones within the same individual, such as ribs, femora, tibiae, or phalanges (Cullen et al., 2014, 2021; D'Emic et al., 2023; Qin et al., 2021). This is a consequence of the absence of medullary cavity expansion, which obscures the early growth record in hollow long bones (Cullen et al., 2014; Erickson, 2014). Again, this pattern contrasts with large‐sized theropods such as *Allosaurus*, *Tyrannosaurus*, and carcharodontosaurids, in which the femora (i.e., the main weight‐bearing bone) preserve the more complete growth record despite its intensive medullary cavity expansion (Bybee et al., 2006; Cullen et al., 2020), although a number of published studies did not agree with this observation (D'Emic et al., 2023; Qin et al., 2021).

Age estimations based on fibulae must consider that the growth intervals are markedly irregular due to their asymmetrical growth, and growth rates and biomechanical stresses actuating on fibulae differ from those weight‐bearing hindlimb bones, which may hinder correspondence between CGMs across the same skeleton. In addition, in the case of *Berthasaura's* fibula (which presents a combination of annuli and LAGs), the annuli in the fibulae do not necessarily reflect an annual cycle. Despite these assumptions, the fibula of MN‐7821‐V preserved one more CGM than the femur and tibia, making this element one of the most suitable bones for age inferences in small ceratosaurs and other small‐sized theropods because it preserves the longer primary tissue growth record (but see Cullen et al., 2014). It is important to note, however, that we found no reason for the preferential use of fibulae over the femur or tibia in age inferences, given that similar age estimates can be obtained by combining age retro‐calculation and CGM counting of hollow bones with wide medullary cavities.

In multi‐element sampling, the simplified shape, asymmetrical growth, and absence of the medullary cavity of some theropod fibula may complicate age retro‐calculation applicable to other long bones and growth‐curves model‐fitting, as it has been suggested by previous authors (e.g., Cullen et al., 2014), which could limit comparisons. Given that estimations based on the femur and tibia resulted in similar ages to the age estimated with fibula, any bias related to the particularity of growth and shape of fibulae can be attenuated with multi‐sampling analyses.

The inter‐element variation in the number of growth marks can underestimate or overestimate individual age based on single elements. The retro‐calculation models reconstructed 3 to 4 years old at the time of death for *Berthasaura* MN‐7821‐V based on tibia and femur, respectively. This suggests that at least one LAG was erased by the medullary cavity expansion in these bones. Interestingly, the cyclical growth marks count of fibula (3 CGM) matches the lower age estimates based on femur and tibia.

Multi‐element analyses in extant crocodilians suggested that despite CGM count exhibiting few intraskeletal variations, growth marks are consistent across skeletons from the same individual (except in the case of extensive medullary cavity expansion), enabling researchers to trace equivalences across the skeleton (Woodward et al., 2014). If there is correspondence in *Berthasaura*'s CGMs, we presume that the two outermost *annuli* of the fibula are chronologically equivalent to both LAGs of the femur and tibia, whereas the innermost LAG of the fibula would be equivalent to the inner erased growth mark of the femur and tibia. If this is correct, the combination of LAG retrocalculation, age estimation, and multi‐element sampling attenuated the effect of histovariability on age inferences in fossil vertebrates.

Our data enable us to make few comments on the retro‐calculation LAG approach. The higher number of estimated CGM in the femur (4) over the tibia (3), despite the same LAG number, likely results from particularly narrower growth intervals of the femur (Table S1) together with a considerably wider medullary cavity (Table 1), which overestimates the age. This observation highlighted a limitation of age retro‐calculations based on metric extrapolation from preserved growth zones. The bias in this method was only identified by correlating different bones together in a multi‐element analysis.

Another important source of histovariability found in *Berthasaura* is the type of bone matrix. The perimedullary cortex of the femur and tibia of *Berthasaura* shared predominantly parallel‐fibered bone matrices. The fibula, however, comprises mostly woven matrix, which alongside primary osteons, features the fibrolamellar complex. This pattern indicates a remarkable level of inter‐skeletal variation in *Berthasaura*, rarely reported among the same body parts of a non‐coelurosaurian theropod.

Among ceratosaurs, multi‐element histological analyses were performed in the unnamed Argentinean abelisaurid MMCh‐PV 69 (Canale et al., 2016), the Malagasy abelisaurid *Majugasaurus crenatissinus* FMNH PR 2278 (D'Emic et al., 2023), and the ceratosaurid *Ceratosaurus nasicornis* BYU 881–12,893 (Sombathy et al., 2025). The specimen MMCh‐PV 69 exhibits a femur composed predominantly of parallel‐fibered bone, similar to abelisaurid and noasaurid femora, but differing from *Berthasaura* MN 7821‐V by having a predominant circumferential arrangement of neurovascular canals (=laminar of Padian & Lamm, 2013). Whereas the dorsal rib of MMCh‐PV 69 consists of woven bone overlain by an external layer of avascular lamellar bone, intercepted by 14 LAGs. In *Majungasaurus*, the sampled bones show substantial variation in bone matrix and number of LAGs. The dorsal rib preserved a completely parallel‐fibered cortex interrupted by 14 LAGs. The femur presents 11 LAGs and is primarily composed of parallel‐fibered bone with portions of lamellar and woven tissues with longitudinal and circumferential patterns of vascularization, in part, resembling bone matrix composition of MN 7821‐V, except for vascularization. *Majungasaurus*' tibia consists of a mixture of woven and parallel‐fibered matrix and exhibits 15 LAGs. Despite vascularization being circumferential‐longitudinal in *Majungasaurus*' tibia, the higher proportion of parallel‐fibered matrix over woven matrix in comparison to femur was surprisingly similar to *Berthasaura*, suggesting a consistent microstructural pattern between the tibiae of these ceratosaur taxa. The fibula of *Majungasaurus* FMNH PR 2278, however, is composed of lamellar and parallel‐fibered tissues with longitudinal vascularization, rather than fibrolamellar and lamellar cortex of MN 7821‐V's fibula. In addition, *Majungasaurus'* fibula exhibits several packs of multi‐LAGs, which differ substantially from the growth record of MN 7821‐V fibula. In *C. nasicornis*, the dorsal rib is primarily of lamellar type, characterized by an intensively remodeled deep cortex. The number of LAGs in this specimen ranges from 12 to 14. *Ceratosaurus*' femur contains a mixture of woven bone, vascular and avascular parallel‐fibered matrices, and a highly vascularized circumferential and radial canal network. This vascularization differs from the reticular‐longitudinal pattern of *Berthasaura*, but resembles the aforementioned abelisaurids.

Bone matrix type varies within the same cross‐section of noasaurid specimens, such as the Malagasy noasaurid *Masiakasaurus* and the Brazilian *Vespersaurus* (de Souza et al., 2020). However, sampling in these cases was based on isolated long bones, which limits the extrapolation to their whole skeletons. Regarding *Masiakasaurus*, the species is characterized by the predominance of parallel‐fibered bone with reticular‐plexiform vascularization in both femur and tibia that suggests a slow growth rate even in early ontogenetic stages, and small portions of fibrolamellar bone (Lee & O'Connor, 2013). Microstructural analyses with ceratosaur femora and tibiae tentatively assigned to *Vespersaurus* from *Cemitério dos Pterossauros* Quarry revealed bones formed almost exclusively by parallel‐fibered bone, except in the fully grown tibiae CP. V 4130 and CP. V 2385, which exhibited small portions of woven‐fibered tissue within predominantly parallel‐fibered bone matrix (de Souza et al., 2020). In addition, *Berthasaura*'s femur and tibia exhibit highly localized osteonal remodeling, which is shared with all sampled ceratosaurs and several other theropods (Bybee et al., 2006; D'Emic et al., 2012; Lee & O'Connor, 2013, and references herein).

In contrast, lower levels of inter‐element variation were reported in the abelisaurids *Aucasaurus* and *Quilmesaurus* (Baiano et al., 2024; Baiano & Cerda, 2017, 2023). Although bone matrix does not vary within the section, this assumption cannot be extrapolated to other bones. Among noasaurids, the only species to show lower intra‐element variation is the Chinese *Limusaurus inextricabilis* and the putative noasaurid femur from the Kem Kem beds (Morocco) ROM 64666, whose cortices are exclusively formed of fibrolamellar tissue (Evans et al., 2015; Wang et al., 2017). The assignment of ROM 64666 within noasaurid is much debatable and will not be discussed here, but *Limusaurus* deserves attention.

Several individuals of *Limusaurus* of different sizes were histologically sampled to assess age and delimit ontogenetic classes (Wang et al., 2017). Fibulae were sectioned in the study, revealing only fibrolamellar bone. It was assumed by de Souza et al. (2020) that *Limusaurus* had a unique bone growth strategy among the clade of low‐growing noasaurids. In parallel, *Limusaurus* is one of the smallest noasaurids known (ca. 1.7 m body length), which would argue against the assumption of parallel‐fibered bone evolving with miniaturization in Ceratosauria (de Souza et al., 2020; contra Aureliano et al., 2023). However, the predominance of fibrolamellar cortex in the fibula of *Berthasaura* (ca. 1 m body length) alongside parallel‐fibered matrices in the femur and tibia suggests that different bone matrices can be exclusive to one bone of the leg. Therefore, fibulae alone may underestimate physiological interpretations in noasaurids. It casts doubt on whether the parallel‐fibered bone is widespread among *Limusaurus*'s skeleton, and only a multi‐element investigation of this taxon can solve this question. Therefore, despite fibulae representing one of the best bones to infer age, it is not the most suitable element to assess metabolic rates and growth dynamics of an individual alone.

Our data supports a considerable level of intra‐skeletal variation among ceratosaurs, especially in bone matrix type. Therefore, taxa that exhibit the lower level of variation, such as *Aucasaurus*, *Quilmesaurus*, and *Limusaurus*, may be an artifact due to under sampling.

### 4.3 Ontogenetic assignment and growth dynamic of *Berthasaura leopoldinae*

The absence of lamellae circumferentiales externae (=external fundamental system) in combination with homogeneous vascularization across the sections of femur, tibia, and fibula indicates that the holotype of *Berthasaura* MN‐7821‐V was not fully grown at the time of death. One could assume that *Berthasaura* had an “undetermined” growth, but the presence of lamellae circumferentiales externae in other noasaurids (such as *Masiakasaurus*, Pterosaur Graveyard noasaurids, and CPPLIP 1490; Lee & O'Connor, 2013; Martinelli et al., 2019; de Souza et al., 2020) makes this assumption less plausible.

In the original description of *Berthasaura* (de Souza et al., 2021), the holotype specimen was regarded as a young sub‐adult based on a mix of immature and mature anatomical traits, such as open neurocentral sutures and well‐ossified articular surfaces of long bones. Consistently, the bone microstructure of MN‐7821‐V revealed a mix of immature and mature histological characteristics. Indicatives of microstructural immaturity are neurovascular network homogeneous across the cortex revealing constant blood supply through life until the time of death; presence of a small portion of fibrolamellar bone indicating fast, sustained bone growth, which is commonly found in earlier ontogenetic stages of archosaurs (Shapiro & Wu, 2019). Indicatives that the individual was close to skeletal maturity are the absence of neurovascular canals opening to the subperiosteal surface, which indicates less active bone deposition; presence of lamellae circumferentiales internae, which correlates to final stages of medullary cavity expansion; presence of secondary remodeling in the inner cortex (although it has been proved to be not exclusive of mature individuals). This mosaic of juvenile and adult histological traits suggests that *Berthasaura*'s holotype was not fully grown nor a young juvenile, that is, a subadult, corroborating what was inferred based on morphology. This is a case of full agreement between osteohistology and morphology.

Comparisons with presumably equivalent ontogenetic stages of *Limusaurus* suggested that *Berthasaura* holotype was equivalent to the ontogenetic Stage IV of the Chinese noasaurid, based exclusively on the degree of fusion between bone sutures (de Souza et al., 2021). The histological data presented here corroborate this assumption, given that fibulae of both MN‐7821‐V (*Berthasaura*, holotype) and IVPP V15923 (*Limusaurus*, stage IV) preserve three cyclical growth marks in fibrolamellar bone cortices. Interestingly, our data reveal that the time of post‐developmental maturation was peculiarly similar among these two ceratosaur species, despite the deep time separating them and the not closely related phylogenetic relationship.

## 5 CONCLUSIONS

This study is the first ontogenetic histological analysis of a single ceratosaur specimen using three different bones, revealing a considerable level of intra‐skeletal variation. Bone histology of *Berthasaura* MN‐7821‐V matches that of other noasaurids and abelisaurid ceratosaurs, mostly represented by slower‐formed bone tissue type parallel‐fibered. It demonstrates that the holotype was a small‐bodied dinosaur about 3–6 years of life, and that skeletal maturity was not attained before death, closely matching with ontogenetic assessment (de Souza et al., 2021) based on ossified synchondroses. There is an appreciable difference in size and bone development trajectories between *Cemitério dos Pterossauros* ceratosaurs (de Souza et al., 2020, 2021). The available histological data endorse the recognition of at least two small‐bodied ceratosaur taxa within this site, corroborating taxonomy based on morphology.

Growth trajectories were curiously similar among *Berthasaura* and *Limusaurus*, despite deep time and phylogenetic distances separating these taxa. This study therefore demonstrates the potential equivocal conclusions associated with single‐bone analyses when attempting detailed metabolic rates or ontogenetic histology‐based interpretations, and the importance of utilizing multiple element samplings when assessing life histories of extinct taxa.

## AUTHOR CONTRIBUTIONS

**Geovane Alves de Souza:** Conceptualization; methodology; investigation; formal analysis; data curation; project administration; writing – review and editing; writing – original draft; visualization; software. **Marina Bento Soares:** Conceptualization; investigation; funding acquisition; writing – original draft; writing – review and editing; methodology; formal analysis; data curation; supervision; resources; visualization; validation. **Luiz Carlos Weinschütz:** Conceptualization; investigation; methodology; data curation; supervision; resources; formal analysis; writing – review and editing. **Alexander Wilhelm Armin Kellner:** Conceptualization; validation; investigation; funding acquisition; writing – review and editing; resources; supervision.

## FUNDING INFORMATION

This study was granted by Coordenação de Aperfeiçoamento de Pessoal de Nível Superior (CAPES#8887314459/2019‐00 to Alexander Wilhelm Armin Kellner), Conselho Nacional de Desenvolvimento Ciêntífico e Tecnológico (CNPq #308515/2023‐4 to Marina Bento Soares, #406779/2021‐0 to Alexander Wilhelm Armin Kellner, INCT Paleovert 406902/2022‐4, to Alexander Wilhelm Armin Kellner and Marina Bento Soares, 408309/2025‐3) and Fundação Carlos Chagas de Amparo à Pesquisa do Estado do Rio de Janeiro (FAPERJ #E‐26/201.095/2022 to Alexander Wilhelm Armin Kellner, E‐26/010.002178/2019, E‐26/204.181/2024 to Marina Bento Soares).

## CONFLICT OF INTEREST STATEMENT

The authors declare no conflicts of interest.

## Supporting information


**Data S1:** Supporting Information.

## References

[ar70110-bib-0001] Aureliano, T. , Ghilardi, A. M. , Fonseca, P. H. , Martinelli, A. G. , & Marinho, T. S. (2023). The evolution and diversification of growth strategies in abelisauroid theropods. Journal of Vertebrate Paleontology, 43(3), e2298395. 10.1080/02724634.2023.2298395

[ar70110-bib-0002] Baiano, M. A. , & Cerda, I. A. (2017). Bone microstructure of *Quilmesaurus curriei* (Theropoda; Abelisauridae). Reunión de Comunicaciones de la Asociación Paleontológica Argentina: Libro de Resúmenes 18:2.

[ar70110-bib-0003] Baiano, M. A. , & Cerda, I. A. (2023). Osteohistology of *Aucasaurus garridoi* (Dinosauria, Theropoda, Abelisauridae): Inferences on lifestyle and growth strategy. Historical Biology, 35(5), 693–704. 10.1080/08912963.2022.2063052

[ar70110-bib-0004] Baiano, M. A. , Cerda, I. A. , Bertozzo, F. , & Pol, D. (2024). New information on paleopathologies in non‐avian theropod dinosaurs: A case study on South American abelisaurids. BMC Ecology and Evolution, 24(1), 6. 10.1186/s12862-023-02187-x PMC10829224 38291378

[ar70110-bib-0005] Botelho, J. F. , Smith‐Paredes, D. , Soto‐Acuna, S. , O'Connor, J. , Palma, V. , & Vargas, A. O. (2016). Molecular development of fibular reduction in birds and its evolution from dinosaurs. Evolution, 70(3), 543–554. 10.1111/evo.12882 PMC5069580 26888088

[ar70110-bib-0006] Buffrénil, V. , Ricqlès, A. J. , Zylberberg, L. , & Padian, K. (2021). Vertebrate skeletal histology and paleohistology (p. 824). CRC Press.

[ar70110-bib-0007] Bybee, P. J. , Lee, A. H. , & Lamm, E.‐T. (2006). Sizing the Jurassic theropod dinosaur Allosaurus: Assessing growth strategy and evolution of ontogenetic scaling of limbs. Journal of Morphology, 267(3), 347–359. 10.1002/jmor.10406 16380967

[ar70110-bib-0008] Canale, J. L. , Cerda, I. , Novas, F. , & Haluza, A. (2016). Small‐sized abelisaurid (Theropoda: Ceratosauria) remains from the Upper Cretaceous of northwest Patagonia, Argentina. Cretaceous Research, 62, 18–28. 10.1016/j.cretres.2016.02.001

[ar70110-bib-0009] Castanet, J. , Francillon‐Vieillot, H. , Meunier, F. J. , & De Ricqles, A. (1993). Bone and individual aging. In B. K. Hall (Ed.), Bone Growth – B (pp. 245–283). CRC Press.

[ar70110-bib-0010] Castanet, J. , & Smirina, E. (1990). Introduction to the skeletochronological method in amphibians and reptiles. Annales des Sciences Naturelles, 11, 191–196.

[ar70110-bib-0011] Chinsamy, A. (1997). Assessing the biology of fossil vertebrates through bone histology. Palaeontologia Africana, 33, 29–35.

[ar70110-bib-0012] Cullen, T. M. , Canale, J. I. , Apesteguía, S. , Smith, N. D. , Hu, D. , & Makovicky, P. J. (2020). Osteohistological analyses reveal diverse strategies of theropod dinosaur body‐size evolution. Proceedings of the Royal Society B: Biological Sciences, 287(1939), 20202258. 10.1098/rspb.2020.2258 PMC7739506 33234083

[ar70110-bib-0013] Cullen, T. M. , Evans, D. C. , Ryan, M. J. , Currie, P. J. , & Kobayashi, Y. (2014). Osteohistological variation in growth marks and osteocyte lacunar density in a theropod dinosaur (Coelurosauria: Ornithomimidae). BMC Evolutionary Biology, 14(1), 231. 10.1186/s12862-014-0231-y PMC4269922 25421260

[ar70110-bib-0014] Cullen, T. M. , Simon, D. J. , Benner, E. K. , & Evans, D. C. (2021). Morphology and osteohistology of a large‐bodied caenagnathid (Theropoda, Oviraptorosauria) from the Hell Creek Formation (Montana): Implications for size‐based classifications and growth reconstruction in theropods. Papers in Palaeontology, 7(2), 751–767. 10.1016/j.cub.2021.06.013

[ar70110-bib-0015] Curry, K. A. (1999). Ontogenetic histology of *Apatosaurus* (Dinosauria: Sauropoda): New insights on growth rates and longevity. Journal of Vertebrate Paleontology, 19(4), 654–665.

[ar70110-bib-0016] de Margerie, E. , Cubo, J. , & Castanet, J. (2002). Bone typology and growth rate: Testing and quantifying ‘Amprino's rule’ in the mallard (*Anas platyrhynchos*). Comptes Rendus Biologies, 325(3), 221–230. 10.1016/s1631-0691(02)01429-4 12017770

[ar70110-bib-0017] de Souza, G. A. , Soares, M. B. , Brum, A. S. , Zucolotto, M. E. , Sayão, J. M. , Weinschütz, L. C. , & Kellner, A. W. (2020). Osteohistology and growth dynamics of the Brazilian noasaurid *Vespersaurus paranaensis* Langer *et al*., 2019 (Theropoda: Abelisauroidea). PeerJ, 8, e9771. 10.7717/peerj.9771 PMC7500327 32983636

[ar70110-bib-0018] de Souza, G. A. , Soares, M. B. , Weinschütz, L. C. , Wilner, E. , Lopes, R. T. , de Araújo, O. M. O. , & Kellner, A. W. A. (2021). The first edentulous ceratosaur from South America. Scientific Reports, 11(1), 22281. 10.1038/s41598-021-01312-4 PMC8602317 34795306

[ar70110-bib-0019] de Souza, G. A. D. , Bulak, B. A. , Soares, M. B. , Sayão, J. M. , Weinschütz, L. C. , Batezelli, A. , & Kellner, A. W. A. (2023). The Cretaceous Neornithine record and new Vegaviidae specimens from the López de Bertodano Formation (Upper Maastrichthian) of Vega Island, Antarctic Peninsula. Anais da Academia Brasileira de Ciências, 95(Suppl 3), e20230802. 10.1590/0001-3765202320230802 38088642

[ar70110-bib-0020] D'Emic, M. D. , Melstrom, K. M. , & Eddy, D. R. (2012). Paleobiology and geographic range of the large‐bodied Cretaceous theropod dinosaur *Acrocanthosaurus atokensis* . Palaeogeography, Palaeoclimatology, Palaeoecology, 333–334, 13–23. 10.1016/j.palaeo.2012.03.003

[ar70110-bib-0021] D'Emic, M. D. , O'Connor, P. M. , Sombathy, R. S. , Cerda, I. , Pascucci, T. R. , Varricchio, D. , Pol, D. , Dave, A. , Coria, R. A. , & Curry Rogers, K. A. (2023). Developmental strategies underlying gigantism and miniaturization in non‐avialan theropod dinosaurs. Science, 379(6634), 811–814. 10.1126/science.adc8714 36821658

[ar70110-bib-0022] Eleutério, L. H. S. , Bantim, R. A. M. , Lima, F. J. , Andrade, R. C. L. P. , Saraiva, A. A. F. , Kellner, A. W. A. , & Sayao, J. M. (2015). Biomechanical and physiological influences on the osteohistological deposition of Anhangueria (Pterosauria, Pterodactyloidea). Revista Brasileira de Paleontologia, 18(3), 403–412.

[ar70110-bib-0023] Enlow, D. H. (1969). The bone of reptiles. In C. Gans , A. Bellairs , & T. Parsons (Eds.), Biology of the Reptilia Volume 1: Morphology A (pp. 45–80). Academic Press.

[ar70110-bib-0024] Enlow, D. H. , & Brown, S. O. (1957). A comparative histological study of fossil and recent bone tissues, Part 2. Texas Journal of Science, 9, 186–214.

[ar70110-bib-0025] Erickson, G. M. (2014). On dinosaur growth. Annual Review of Earth and Planetary Sciences, 42, 675–697. 10.1146/annurev-earth-060313-054858

[ar70110-bib-0026] Erickson, G. M. , Rauhut, O. W. M. , Zhou, Z. , Turner, A. H. , Inouye, B. D. , Hu, D. , & Norell, M. A. (2009). Was dinosaurian physiology inherited by birds? Reconciling slow growth in *Archaeopteryx* . PLoS One, 4(10), e7390. 10.1371/journal.pone.0007390 PMC2756958 19816582

[ar70110-bib-0027] Esteban, M. , Castanet, J. , & Sanchiz, B. (1998). Inferring age and growth from remains of fossil and predated recent anurans: A test case using skeletochronology. Canadian Journal of Zoology, 76(9), 1689–1695.

[ar70110-bib-0028] Evans, D. C. , Barrett, P. M. , Brink, K. S. , & Carrano, M. T. (2015). Osteology and bone microstructure of new, small theropod dinosaur material from the early Late Cretaceous of Morocco. Gondwana Research, 27(3), 1034–1041.

[ar70110-bib-0029] Farias, B. D. M. , Desojo, J. B. , Cerda, I. A. , Ribeiro, A. M. , Ferigolo, J. , Carlisbino, T. , Schultz, C. L. , Mastrantonio, B. M. , & Soares, M. B. (2024). Bone histology supports gregarious behavior and an early ontogenetic stage to *Decuriasuchus quartacolonia* (Pseudosuchia: Loricata) from the Middle‐Late Triassic of Brazil. The Anatomical Record, 307(4), 957–973. 10.1002/ar.25365 38088505

[ar70110-bib-0030] Francillon‐Vieillot, H. , Arntzen, J. W. , & Geraudie, J. (1990). Age, growth and longevity of sympatric *Triturus cristatus*, *T. marmoratus* and their hybrids (Amphibia, Urodela): A skeletochronological comparison. Journal of Herpetology, 24(1), 13–22. 10.2307/1564284

[ar70110-bib-0031] Goldman, H. M. , McFarlin, S. C. , Cooper, D. M. , Thomas, C. D. L. , & Clement, J. G. (2009). Ontogenetic patterning of cortical bone microstructure and geometry at the human mid‐shaft femur. The Anatomical Record, 292(1), 48–64. 10.1002/ar.20778 19051245

[ar70110-bib-0032] Grady, J. M. , Enquist, B. J. , Dettweiler‐Robinson, E. , Wright, N. A. , & Smith, F. A. (2014). Evidence for mesothermy in dinosaurs. Science, 344(6189), 1268–1272. 10.1126/science.1253143 24926017

[ar70110-bib-0033] Griffin, C. T. (2018). Pathological bone tissue in a Late Triassic neotheropod fibula, with implications for the interpretation of medullary bone. New Jersey State Museum Investigations, 6, 2–10.

[ar70110-bib-0034] Horner, J. R. , de Ricqlès, A. , & Padian, K. (1999). Variation in dinosaur skeletochronology indicators: Implications for age assessment and physiology. Paleobiology, 25(3), 295–304. 10.1017/S0094837300021308

[ar70110-bib-0035] Horner, J. R. , de Ricqlès, A. , & Padian, K. (2000). Long bone histology of the hadrosaurid dinosaur Maiasaura peeblesorum: Growth dynamics and physiology based on an ontogenetic series of skeletal elements. Journal of Vertebrate Paleontology, 20(1), 115–129. 10.1671/0272-4634(2000)020[0115:LBHOTH]2.0.CO;2

[ar70110-bib-0036] Horner, J. R. , & Goodwin, M. B. (2009). Extreme cranial ontogeny in the Upper Cretaceous dinosaur Pachycephalosaurus. PLoS One, 4(10), e7626. 10.1371/journal.pone.0007626 PMC2762616 19859556

[ar70110-bib-0037] Horner, J. R. , & Padian, K. (2004). Age and growth dynamics of Tyrannosaurus rex. Proceedings of the Royal Society of London. Series B, Biological Sciences, 271(1551), 1875–1880. 10.1098/rspb.2004.2829 PMC1691809 15347508

[ar70110-bib-0038] Hübner, T. R. (2012). Bone histology in *Dysalotosaurus lettowvorbecki* (Ornithischia: Iguanodontia)—variation, growth, and implications. PLoS One, 7, e29958. 10.1371/journal.pone.0029958 PMC3253128 22238683

[ar70110-bib-0040] International Committees on Veterinary Gross Anatomical Nomenclature . (1994). Nomina Anatomica Veterinaria (4th edn), the revised (2nd edn) of Nomina Histologica Veterinaria and Nomina Embryologica Veterinaria, Zürich and Ithaca, New York.

[ar70110-bib-0041] Kellner, A. W. , Weinschütz, L. C. , Holgado, B. , Bantim, R. A. , & Sayao, J. M. (2019). A new toothless pterosaur (Pterodactyloidea) from Southern Brazil with insights into the paleoecology of a Cretaceous desert. Anais da Academia Brasileira de Ciencias, 91(Suppl 2), e20190768. 10.1590/0001-3765201920190768 31432888

[ar70110-bib-0042] Klein, N. , & Sander, M. (2008). Ontogenetic stages in the long bone histology of sauropod dinosaurs. Paleobiology, 34(2), 247–263. 10.1666/0094-8373(2008)034[0247:OSITLB]2.0.CO;2

[ar70110-bib-0043] Lamm, E. T. (2007). Paleohistology widens the field of view in paleontology. Proceedings, Microscopy and Microanalysis, 13(S02), 50.

[ar70110-bib-0044] Langer, M. C. , Martins, N. O. , Manzig, P. C. , Ferreira, G. S. , Marsola, J. C. A. , Fortes, E. , Lima, R. , Sant'ana, L. C. F. , Vidal, L. S. , & Lorençato, S. R. H. (2019). A new desert‐dwelling dinosaur (Theropoda, Noasaurinae) from the Cretaceous of south Brazil. Scientific Reports, 9(1), 1–31. 10.1038/s41598-019-45306-9 PMC6594977 31243312

[ar70110-bib-0045] Leclair, R. (1990). Relationships between relative mass of the skeleton, endosteal resorption, habitat and precision of age determination in ranid amphibians. Annales des Sciences Naturalles, 11, 205–209.

[ar70110-bib-0046] Lee, A. H. , & O'Connor, P. M. (2013). Bone histology confirms determinate growth and small body size in the noasaurid theropod Masiakasaurus knopfleri. Journal of Vertebrate Paleontology, 33(4), 865–876. 10.1080/02724634.2013.743898

[ar70110-bib-0047] Lee, A. H. , & Werning, S. (2008). Sexual maturity in growing dinosaurs does not fit reptilian growth models. Proceedings of the National Academy of Sciences of the United States of America, 105(2), 582–587. 10.1073/pnas.070890310 PMC2206579 18195356

[ar70110-bib-0048] Manzig, P. C. , Kellner, A. W. A. , Weinschütz, L. C. , Fragoso, C. E. , Veja, C. S. , Guimarães, G. B. , Godoy, L. C. , Liccardo, A. , Ricetti, J. H. Z. , & De Moura, C. C. (2014). Discovery of a rare pterosaur bone bed in a Cretaceous desert with insights on ontogeny and behavior of flying reptiles. PLoS One, 9(8), 100005. 10.1371/journal.pone.0100005 PMC4131874 25118592

[ar70110-bib-0049] Martinelli, A. G. , Marinho, T. S. , Egli, F. B. , Hechenleitner, E. M. , Iori, F. V. , Veiga, F. H. , Basilici, G. , Soares, M. V. T. , Marconato, A. , & Ribeiro, L. C. B. (2019). Noasaurid theropod (Abelisauria) femur from the Upper Cretaceous Bauru Group in Triângulo Mineiro (Southeastern Brazil). Cretaceous Research, 104, 104181. 10.1016/j.cretres.2019.07.011

[ar70110-bib-0050] Mitchell, J. , & Sander, P. M. (2014). The three‐front model: A developmental explanation of long bone diaphyseal histology of Sauropoda. Biological Journal of the Linnean Society, 112(4), 765–781. 10.1111/bij.12324

[ar70110-bib-0051] Mitchell, J. , Sander, P. M. , & Stein, K. (2017). Can secondary osteons be used as ontogenetic indicators in sauropods? Extending the histological ontogenetic stages into senescence. Paleobiology, 43(2), 321–342. 10.1017/pab.2016.47

[ar70110-bib-0052] Mitchell, J. , & Van Heteren, A. H. (2016). A literature review of the spatial organization of lamellar bone. Compte Rendus Palevol, 15(1–2), 23–31. 10.1016/j.crpv.2015.04.007

[ar70110-bib-0053] Padian, K. , de Ricqlès, A. J. , & Horner, J. R. (2001). Dinosaurian growth rates and bird origins. Nature, 412(6845), 405–408. 10.1038/35086500 11473307

[ar70110-bib-0054] Padian, K. , & Lamm, E.‐T. (2013). Bone Histology of Fossil Tetrapods: Advancing Methods, Analysis, and Interpretation (p. 285). University of California Press.

[ar70110-bib-0055] Peabody, E. E. (1961). Annual growth zones in vertebrates (living and fossil). Journal of Morphology, 108, 11–62.

[ar70110-bib-0056] Prondvai, E. (2017). Medullary bone in fossils: Function, evolution and significance in growth curve reconstructions of extinct vertebrates. Journal of Evolutionary Biology, 30(3), 440–460. 10.1111/jeb.13019 27862520

[ar70110-bib-0057] Prondvai, E. , Stein, K. H. , de Ricqlès, A. , & Cubo, J. (2014). Development‐based revision of bone tissue classification: The importance of semantics for science. Biological Journal of the Linnean Society, 112(4), 799–816. 10.1111/bij.12323

[ar70110-bib-0058] Qin, Z. , Zhao, Q. , Choiniere, J. N. , Clark, J. M. , Benton, M. J. , & Xu, X. (2021). Miniaturization and Phylogenetic Radiation in Alvarezsauroids: Insights from histological analysis. Current Biology, 31(16), 3687–3693.e5. 10.1016/j.cub.2021.06.013 34233160

[ar70110-bib-0059] Ricqlès, A. J. (1969). Recherches paléohistologiques sur les os longs des Tétrapodes. II, Quelques observations sur la structure des longs des Thériodontes. Annales de Paléontologie, 55(61), 51–129.

[ar70110-bib-0060] Ricqlès, A. J. (1974). Evolution of endothermy: Histological evidence. Evolutionary Theory, 1(2), 51–80.

[ar70110-bib-0061] Rolando, M. A. , Cerroni, M. A. , Marsà, J. A. G. , Motta, M. J. , Rozadilla, S. , Eglí, F. B. , & Novas, F. E. (2021). A new medium‐sized abelisaurid (Theropoda, Dinosauria) from the late cretaceous (Maastrichtian) Allen Formation of Northern Patagonia, Argentina. Journal of South American Earth Sciences, 105, 102915. 10.1016/j.jsames.2020.102915

[ar70110-bib-0062] Sander, P. M. (2000). Long bone histology of the Tendaguru sauropods: Implications for growth and histology. Paleobiology, 26(3), 466–488. 10.1666/0094-8373(2000)026<>2.0.CO;2

[ar70110-bib-0063] Sayão, J. M. (2003). Histovariability in bones of two pterodactyloid pterosaurs from the Santana Formation, Araripe Basin, Brazil: Preliminary results. In E. Buffetaut & J. M. Mazin (Eds.), Evolution and palaeobiology of pterosaurs (pp. 335–342. (Special Publication 217)). Geological Society. 10.1144/GSL.SP.2003.217.01.21

[ar70110-bib-0064] Shapiro, F. , & Wu, J. Y. (2019). Woven bone overview: structural classification based on its integral role in developmental, repair and pathological bone formation throughout vertebrate groups. European Cells and Matererials Journal, 38, 137–167. 10.22203/ecm.v038a11 31571191

[ar70110-bib-0065] Simões, T. R. , Wilner, E. , Caldwell, M. W. , Weinschütz, L. C. , & Kellner, A. W. (2015). A stem acrodontan lizard in the Cretaceous of Brazil revises early lizard evolution in Gondwana. Nature Communications, 6(1), 8149. 10.1038/ncomms9149 PMC4560825 26306778

[ar70110-bib-0066] Sombathy, R. , O'Connor, P. M. , & D'Emic, M. D. (2025). Osteohistology of the unusually fast‐growing theropod dinosaur *Ceratosaurus* . Journal of Anatomy, 247, 765–789. 10.1111/joa.14186 PMC12397097 39909856

[ar70110-bib-0067] Stein, K. , & Sander, P. M. (2009). Histological core drilling: a less destructive method for studying bone histology. In M. A. Brown , J. F. Kane , & W. G. Parker (Eds.), Methods in fossil preparation: proceedings of the first annual Fossil Preparation and Collections Symposium (pp. 69–80). Petrified Forest National Park.

[ar70110-bib-0068] Tutken, T. , Pfretzschner, H.‐U. , Vennemann, T. W. , Sun, G. , & Wang, Y. D. (2004). Paleobiology and skeletochronology of Jurassic dinosaurs: implications from the histology and oxygen isotope compositions of bone. Palaeogeography, Palaeoclimatology, Palaeoecology, 206(3–4), 217–238. 10.1016/j.palaeo.2004.01.005

[ar70110-bib-0069] Varricchio, D. J. (1993). Bone microstructure of the Upper Cretaceous theropod dinosaur *Troodon formosus* . Journal of Vertebrate Paleontology, 13(1), 99–104. 10.1080/02724634.1993.10011490

[ar70110-bib-0070] Wang, S. , Stiegler, J. , Amiot, R. , Wang, X. , Du, G. , Clark, J. M. , & Xu, X. (2017). Extreme ontogenetic changes in a ceratosaurian theropod. Current Biology, 27(1), 144–148. 10.1016/j.cub.2016.10.043 28017609

[ar70110-bib-0071] Waskow, K. , & Sander, P. M. (2014). Growth record and histological variation in the dorsal ribs of *Camarasaurus* sp. (Sauropoda). Journal of Vertebrate Paleontology, 34(4), 852–869. 10.1080/02724634.2014.840645

[ar70110-bib-0072] Woodward, H. N. (2019). *Maiasaura* (Dinosauria: Hadrosauridae) Tibia Osteohistology reveals non‐annual cortical vascular rings in young of the year. Frontiers in Earth Science, 7, 50. 10.3389/feart.2019.00050

[ar70110-bib-0073] Woodward, H. N. , Horner, J. R. , & Farlow, J. O. (2011). Osteohistological evidence for determinate growth in the American alligator. Journal of Herpetology, 45(3), 339–342. 10.1670/10-274.1

[ar70110-bib-0074] Woodward, H. N. , Horner, J. R. , & Farlow, J. O. (2014). Quantification of intraskeletal histovariability in *Alligator mississippiensis* and implications for vertebrate osteohistology. PeerJ, 2, e422. 10.7717/peerj.422 PMC4060058 24949239

