# Supplementary material for: Growth dynamics, skeletochronology, and histovariability of the theropod dinosaur Berthasaura leopoldinae
Source: Anat Rec (Hoboken). 2025 Dec 15;309(11):3169–86. doi: 10.1002/ar.70110 (PMC13644900; doi:10.1002/ar.70110)
Supplement: Supplementary file 1 [file AR-309-3169-s001.docx]

**SUPPLEMENTARY DATA**

**Contents:**

**1. Skeletochronology**

**2. Supplementary figures**

**Supplementary figure 1 – Whole cross section of left femur MN 7821-V.** Lateral points to the left and posterior points to the bottom. Scale bar equals 0.5 mm.

**Supplementary figure 2 – Whole cross section of left tibia MN 7821-V.** Lateral points to the left and posterior points to the bottom. Scale bar equals 1 mm.

**Supplementary figure 3 – Whole cross section of left fibula MN 7821-V.** Lateral points to the left and posterior points to the bottom. Scale bar equals 0.4 mm.

**3. Supplementary references**

**1. Skeletochronology**

Age estimation for MN 7821 V was produced by accounting lines of arrested growth (LAGs). However, LAGs deposited in the earlier ontogeny are commonly erased by medullary cavity expansion, and then this missing growth record must be estimated. The number of missing LAGs can be extrapolated from preserved LAGs (i.e. LAGs retro-calculation). LAG retrocalculation was modified from Horner and Padian (2004). For this purpose, the thickness of each preserved growth zone (cortex deposited between adjacent LAGs) was digitally measured using ImageJ Program. Because the growth zones vary in thickness through the section, we taken 10 measures (radius), in equidistant sites over the entire circumferential extent of each growth zone to obtain average growth zone thicknesses (Supplementary Table 1).

**Supplementary Table 1.** Femur and tibia growth zones widths (in mm).

|  | **Femur** | | | **Tibia** | | |
| --- | --- | --- | --- | --- | --- | --- |
| **Growth zone** | Innermost | Intermediate | Outermost | Innermost | Intermediate | Outermost |
|  | 0.42 | 0.55 | 0.15 | 0.45 | 1.61 | 0.06 |
|  | 0.43 | 0.69 | 0.10 | 0.58 | 1.82 | 0.07 |
|  | 0.44 | 0.72 | 0.14 | 0.62 | 1.89 | 0.07 |
|  | 0.47 | 0.67 | 0.13 | 0.47 | 2.07 | 0.06 |
|  | 0.49 | 0.59 | 0.09 | 0.44 | 1.61 | 0.03 |
|  | 0.42 | 0.58 | 0.11 | 0.46 | 1.43 | 0.06 |
|  | 0.63 | 0.67 | 0.18 | 0.51 | 1.29 | 0.09 |
|  | 0.68 | 0.71 | 0.15 | 0.34 | 1.36 | 0.10 |
|  | 0.51 | 1.06 | 0.17 | 0.48 | 1.32 | 0.10 |
|  | 0.52 | 1.73 | 0.08 | 0.61 | 1.25 | 0.10 |
| **Mean** | 0.50 | 0.79 | 0.13 | 0.49 | 1.56 | 0.07 |
| **Stand Dev** | 0.08 | 0.35 | 0.03 | 0.08 | 0.28 | 0.02 |

Abbreviations: Stand dev = Standard deviation.

After, femur and tibia centroids were calculated on the whole cross-section images (Supplementary Figures 1 – 3), using the polygon tool of ImageJ program. The intercept between X and Y axes were assumed as bone centroids. Then, the distance between the centroid and the innermost LAG was measured at different sides of the sections (anterior, medial, lateral and posterior), totaling four measures by bone. Note that these measures correspond to radius of both empty medullary cavity and bone (primary or secondary), then assumed as missing cortex. Cracked cortical borders were avoided to prevent overestimations. The average radius of missing bone was obtained for femur and tibia (Supplementary Table 2).

Size or weight at birth are the smallest mass/size in the ontogeny, add important biological information and realism by constraining the estimation at the lower end (Grady et al. 2014). Because no LAG is expected to be formed during egg development, we discounted the neonatal femoral/tibial radius to the missing cortices. However, our data consists of a single subadult individual. Thus, we assumed the femur/tibia radius of the hatchling as ca. 1.97 mm based on egg estimation and size of a larger, presumably mature individual of *Berthasaura* (personal communication based on unreported femoral fragment CP.V 8662, in prep.). Finally, the missing growth record assumed here is the empty space of the medullary cavity + partially eroded innermost cortex radius (average) subtracted by the hatchling radius (1.95 mm for femur and 1.48 mm for tibia).

**Supplementary Table 2.** X and Y axes of the cross section at the centroid and thickness of bone lost by medullary cavity expansion (radius), estimate hatchling radius. All measures in millimeters.

| **Bone** | **X** | **Y** | **Missing and partially eroded innermost cortex radius (average)** | **Stand Dev** | **Hatchling radius** | **Missing growth record** |
| --- | --- | --- | --- | --- | --- | --- |
| **Femur** | 6.17 | 4.75 | 3.95 | 1.11 | 1.97 | 1.98 |
| **Tibia** | 5.87 | 4.39 | 3.48 | 0.76 | 1.97 | 1.51 |

Abbreviations: Stand dev = Standard deviation.

Finally, the lost growth record was estimated using theorical models proposed by Horner and Padian (2004). Originally, Horner and Padian (2004) listed five theoretical models: maximum, penultimate, mean, incremental factor, and parabolic. In maximum, penultimate and mean models, the lost growth record is rationed by the thicker growth zone – usually the innermost (Maximum Model); the penultimate growth zone in the case of the later was exceptionally narrowed (Penultimate Model); or by the average thickness of the growth zones (Mean Model). Incremental Factor Model considers a mean percentage increase in the width of LAG intervals, moving centripetally, whereas in the Parabolic Model, hypothesizing that LAG intervals were maximal at the innermost preserved LAG, the intervals of the series progressing centrifugally were applied to the missing centripetal series, mirrored.

The particularly narrowed outermost and innermost growth zones of MN 7821-V, however, underestimate the thickness of the retro-calculated growth zones. Thus, we consider that the Mean, Incremental Factor, and Parabolic models of Horner and Padian (2004) did not fit in with our data because they are underestimated by peculiarly narrow growth zones of MN 7821-V. The only minimally reliable values considered were those obtained from Maximum and Penultimate models, given that the only complete growth zone preserved in MN 7821-V is the penultimate interval. The results from all models are presented in the Supplementary Table 3.

**Supplementary Table 3.** Retro-calculated lines of arrested growth using different models.

| **Bone** | **Maximum Model** | **Penultimate Model** | **Mean Model** | **Incremental factor** | **Parabolic** |
| --- | --- | --- | --- | --- | --- |
| Femur | 2 | 2 | 4 | 0.19* | 3 |
| Tibia | <1 | <1 | <1 | 0.03* | 1 |

*Innermost preserved growth intervals were discarded during the calculation due to their incompleteness.

**Supplementary Figure 1**

**Supplementary Figure 2**

**Supplementary Figure 3**

**Supplementary References**

Grady, J.M., Enquist, B.J., Dettweiler-Robinson, E., Wright, N.A. & Smith, F.A. (2014) Evidence for mesothermy in dinosaurs. *Science* 344 (6189), 1268 –1272.

[DOI: 10.1126/science.1253143](https://doi.org/10.1126/science.1253143)

Horner, J.R. & Padian, K. (2004) [Age and growth dynamics of *Tyrannosaurus rex*](https://doi.org/10.1098%2Frspb.2004.2829). *Proceedings of the Royal Society B: Biological Sciences* 271 (1551), 1875–1880.

<https://doi.org/10.1098/rspb.2004.2829>
